# Multi-frequency passive and active microrheology with optical tweezers

**DOI:** 10.1038/s41598-021-93130-x

**Published:** 2021-07-06

**Authors:** Randhir Kumar, Valerio Vitali, Timo Wiedemann, Robert Meissner, Paolo Minzioni, Cornelia Denz

**Affiliations:** 1 Institute of Applied Physics and Center for Nonlinear Science (CeNoS), University of Muenster, Muenster, 48149 Germany; 2 Department of Electrical, Computer and Biomedical Engineering, University of Pavia, 27100 Pavia, Italy; 3 Present Address: Currently at Optoelectronics Research Centre, University of Southampton, Southampton, Hampshire SO17 1BJ UK

**Keywords:** Biological techniques, Biophysics, Optics and photonics, Physics

## Abstract

Optical tweezers have attracted significant attention for microrheological applications, due to the possibility of investigating viscoelastic properties in vivo which are strongly related to the health status and development of biological specimens. In order to use optical tweezers as a microrheological tool, an exact force calibration in the complex system under investigation is required. One of the most promising techniques for optical tweezers calibration in a viscoelastic medium is the so-called active–passive calibration, which allows determining both the trap stiffness and microrheological properties of the medium with the least a-priori knowledge in comparison to the other methods. In this manuscript, we develop an optimization of the active–passive calibration technique performed with a sample stage driving, whose implementation is more straightforward with respect to standard laser driving where two different laser beams are required. We performed microrheological measurements over a broad frequency range in a few seconds implementing an accurate multi-frequency driving of the sample stage. The optical tweezers-based microrheometer was first validated by measuring water, and then exemplarily applied to more viscous medium and subsequently to a viscoelastic solution of methylcellulose in water. The described method paves the way to microrheological precision metrology in biological samples with high temporal- and spatial-resolution allowing for investigation of even short time-scale phenomena.

## Introduction

Biomechanical properties of living cells and tissues play a crucial role in many vital cell functions such as cell division, cell migration, cellular differentiation, tissue homeostasis and patterning^1–6^. Among these properties, viscoelasticity plays a pivotal role in allowing cells and tissues to undergo proper shape changes and interact with one another^7,8^. The cellular environment consists of a complex fluid that exhibits both viscous and elastic properties. The rheological properties of a complex fluid are represented by the complex shear modulus, which is the ratio of stress over strain and represents the resistance of a fluid/material against deformations. There are a plethora of techniques available to probe these properties on micro-scale level, including atomic force microscopy^9,10^, magnetic twisting cytometry^11^, particle tracking microrheology^12,13^, and optical tweezers^14–16^ (OT). Most of these techniques rely on studying the position of a micro- or nano-sized probe particle. If the probe particle’s motion due to thermal fluctuation is monitored, the approach is called passive microrheology^17,18^. If the probe particle’s motion response under external perturbation is monitored, it is termed active microrheology^19–21^. OT is one of the most widely used techniques for microrheology in recent decades. In order to determine rheological properties, OT requires precise calibration. There are different passive and active calibration methods available in literature^22–26^. However, in complex materials, the most noteworthy among all calibration methods is the so-called *active–passive calibration* (APC) technique^27,28^. It requires the least a-priori knowledge while yielding information about the strength of the optical trap (trap stiffness) and the rheological properties of the analyzed viscoelastic medium. As the name suggests, the APC technique combines passive and active measurements. The external perturbation in the active method can be achieved by modulating either the trapping laser (laser driving) or the sample stage (stage driving).

Active measurements are repeated at different frequencies in order to derive a complete characterization of the system under study and of its biomechanical parameters. Using standard approaches, scanning of consecutive frequencies can take up to several minutes, thus limiting the temporal resolution of this technique. As a consequence, using this approach, measurements taken at different frequencies on a biological sample may be attributed to different cell states, thereby not allowing to obtain a reliable characterization of a specific cell configuration. To solve this issue, multiplexing approaches^29,30^ have been proposed, allowing to increase the frequency-scan speed significantly. APC is commonly performed using a laser-driving approach, where two different trapping and detection laser beams are required. The use of an acoustic optic deflector (AOD) in laser driving allows achieving oscillation frequencies higher than those achievable by stage driving, where piezo-stages are usually employed. However, instead of AOD, a spatial light modulator (SLM) can also be used to modulate the trapping laser^31^. Although, the modulation rate of SLMs is slower than that of AODs, SLMs can also be used because of their relatively simple implementation, especially if a high speed camera, instead of a position-sensitive photodiode, is employed to track the particle position. Nonetheless, the simplicity of a single laser setup makes the implementation of the stage-driving APC generally easier than the laser-driving counterpart. Besides laser and stage driving methods, tracking of optically trapped microbeads with high speed cameras and image analysis^31–33^ have been used in microrhelogy over broad frequency range. Despite the recent advancements in cameras technology^34^, the typical upper limit of the detection rate is still in the order of kHz^35^, while position-sensitive photodiodes can reach rates of the order of MHz^35^, which allows to achieve a better temporal resolution in the tracking of the microbead position.

In this work, we present an optimization of the stage-driving APC technique, that allows measuring viscoelastic properties over a broad frequency range and with high temporal and spatial resolution. A novel algorithm for designing the APC multiplexed driving signal is presented, with special emphasis on the choice of the phase used for each frequency. We also compare measurements with state-of-the-art single frequency signals and multi-frequency signals in the calibration of standard silica microbeads in water. As a system validation, we report the measurement results obtained considering a viscoelastic material (a water-methylcellulose solution) and we show the rheological sensitivity of the calibration in measuring the viscous and elastic contributions. At the end of the paper, we discuss how the choice of optimized experimental parameters allows increasing the calibration bandwidth.

## Theoretical background

Calibration of OT in viscous media can be performed using a purely passive method, such as the power spectrum method^36^. In this case, the thermal motion of a trapped microbead is recorded, which allows calculating the power spectrum $P_U (\omega )$ as given by Eq. 1, where ${\tilde{x}}_U$ is the Fourier transform of the microbead position (footer U stands for undriven and it is used to stress that the bead is not driven by any externally applied force), $\omega$ is the angular frequency and $\langle \rangle$ denotes the averaging operation over the measurement time. A detailed description of the APC theory can be found in Fischer et al.^27^

1 $$\begin{aligned} P_U (\omega ) = \Big \langle | {\tilde{x}}_U(\omega ) |^2 \Big \rangle \end{aligned}$$

If the microbead properties such as radius and density and the medium property such as viscosity are known, it is possible to derive the trap stiffness $\kappa _{PC}$ from the power spectrum, where PC indicates the passive calibration approach. However, when a viscoelastic medium of unknown rheological behavior is considered, the observation of the Brownian fluctuation is not sufficient for the system calibration. In the case of a viscoelastic material, the OT system can be calibrated by taking advantage of the APC technique. APC is a two steps technique. The first (passive) step consists of the determination of the thermal power spectrum, as defined by Eq. 1. The second (active) step is the detection of the trapped microbead response under the influence of an external applied force. One way to apply this external force is to modulate the piezo stage on which the sample is mounted (stage driving). In this case, the active power spectrum ${\tilde{R}}_S(\omega )$ is given by Eq. 2, where ${\tilde{x}}_{dr} (\omega )$ is the Fourier transform of the microbead position in response to the external force and ${\tilde{x}}_{s} (\omega )$ is the Fourier transform of the sample stage position.

2 $$\begin{aligned} {\tilde{R}}_S (\omega ) = \frac{{\tilde{x}}_{dr} (\omega )}{-i \omega {\tilde{x}}_{s} (\omega )} \end{aligned}$$

By combining the passive and the active steps, it is possible to determine the trap stiffness $\kappa _{\omega }$ for a given oscillation angular frequency $\omega$, which is given by Eq. 3. Equation 3 is termed as *calibration equation*, where $k_{B}$ is the Boltzmann constant, *T* is the absolute temperature (in Kelvin), *m* is the mass of trapped microbead and *Re* indicates the real part of active power spectrum.

3 $$\begin{aligned} (\kappa - \omega ^2m)_{\omega } = 2k_BT \cdot \frac{Re({\tilde{R}}_S (\omega ))}{P_U (\omega )} \end{aligned}$$

Once the trap stiffness has been determined, it is possible to derive the viscoelastic properties of the surrounding medium in which the microbead is trapped. The rheological properties can be described by the *friction retardation spectrum* which is defined as ${\tilde{\gamma }}(\omega ) = {\tilde{\gamma }}'(\omega ) + i \omega {\tilde{\gamma }}''(\omega )$, where the real part accounts for dissipative contributions, while the imaginary part accounts for elastic contributions. In case of stage driving, the friction retardation spectrum is given by the following equation:

4 $$\begin{aligned} {\tilde{\gamma }}(\omega ) = \frac{{\tilde{R}}_S (\omega )}{1-i \omega {\tilde{R}}_S (\omega )} \cdot (\kappa - \omega ^2m) \end{aligned}$$

For spherical microbeads of known radius *R* suspended in an incompressible fluid, the friction retadation spectrum can be used to obtain the complex modulus $G^*(\omega )$^18,37,38^:

5 $$\begin{aligned} G^*(\omega ) = \frac{i \omega {\tilde{\gamma }}(\omega )}{6\pi R} \end{aligned}$$

As for the friction retardation spectrum, the complex modulus is a frequency-dependent complex quantity, $G^*(\omega ) = G'(\omega ) + i G''(\omega )$, whose real part (*storage modulus)* accounts for the elastic processes while its imaginary part (*loss modulus*) is related to the dissipative contributions.

The combination of the passive and active measurements is only possible under the Onsager’s regression hypothesis^39^, that is a consequence of the fluctuation–dissipation theorem (FDT)^40^: the microbead displacement caused by the external perturbation has to be comparable with the displacement caused by thermal fluctuation in the undriven system.

## Results

### Multi-frequency driving signal generation

When the APC technique was theoretically presented by Fischer et al.^27^, a sinusoidal signal for each measured frequency was proposed as a driving signal for the active part. The reasons for such a choice were mainly two: (1) a non-sinusoidal driving is more challenging to be experimentally implemented, (2) larger statistical and non-statistical errors were generally introduced by non-sinusoidal driving signals with respect to sinusoidal signals at equal measuring time. The major drawback of sinusoidal driving is that many frequencies have to be measured consecutively, one at a time, in order to reconstruct the rheological parameters over a wide frequency range. This significantly increases the measurement time, thus allowing to obtain information only about the “average” microrheological properties of the sample in a given time interval. Moreover, if the measuring time is too long and measurements on live samples (e.g., cells, bacteria) are carried out, the living organism has time to reorganize its structure, implying the loss of thermodynamic equilibrium and a larger uncertainty in the estimated mechanical properties^41^. Despite the uncertainty increase, the multi-frequency driving signals method has been used to drastically reduce the measuring time to perform measurements in biological samples^29,30^. Additionally, reducing the measurement time also helps to reduce the risk of damaging the biological samples because of the laser-induced heating. On the other hand, the multiplexed signal has to be carefully built up in order to keep the driving amplitude relatively small due to various constraints explained in the following. The microbead position must remain in the linear response region of the optical trap and in the linear detection range of the detection system. Moreover, the external perturbation cannot violate the Onsager’s regression hypothesis, and as a consequence, the maximum amplitude of the microbead oscillation $A_{p,max} = (k_BT / \kappa )^{1/2}$, given by the trap half-width^28^, is generally set as an upper limit. The last constrain implies that the amplitude of the microbead displacement under external perturbation should be as low as possible. For all the experimental results reported in this paper, the amplitude of the microbead oscillation never exceeds 10 nm.

The determination of the maximum amplitude of a multi-frequency signal depends upon the amplitudes and phases of the total sum of all the signals. Thus, amplitudes and phases must be carefully designed. In this section, we present different methods reported in the literature for the generation of a multi-frequency driving signal and we demonstrate how our *iterative phase optimization* method outperforms the others. A multi-frequency driving signal can be generated as a superposition of *N* sinusoidal signals:

6 $$\begin{aligned} S_N (t, \phi _{1,...,N}) = \sum \limits _{i=1}^N A_i \cdot sin(2 \pi f_i t + \phi _i) \end{aligned}$$

where $f_i$ is the *i*th driving frequency, $A_i$ and $\phi _i$ are the corresponding amplitude and phase, respectively. In order to avoid cross-talk in the frequency space, a set of prime numbers can be chosen as driving frequencies, as suggested by Blehm et al.^29^. The amplitudes $A_i$ are independently set for each frequency in order to take into account the frequency dependent response of the trapped microbead subjected to the stage driving^28^. By considering a superimposition of sinusoidal functions, all the amplitudes add up and the maximum amplitude $A_{max}$, which is usually an increasing function of *N*, is defined as:

7 $$\begin{aligned} A_{max}(S_N) = max(S_N(t, \phi _{1,...,N})) - min(S_N(t, \phi _{1,...,N})) \end{aligned}$$

Obviously, a larger number of driving frequencies is desirable in order to obtain a rheological characterization over a wide frequency range. In order to include as many frequencies as possible in the driving signal $S_N$, the phase distribution $\phi _i$ must be chosen to minimize the value of the maximum amplitude $A_{max}(S_N)$. The impact of three different phase distributions on the maximum amplitude was studied by Deng et al.^42^, where a comparison between the following signal phase designs has been carried out:

8 $$\begin{aligned}&Zero \ phase:\qquad \phi _i = 0 \qquad i=1,2,...,N \end{aligned}$$

9 $$\begin{aligned}&Random \ phase:\qquad \phi _i = 2 \pi \cdot rand(0,1) \qquad i=1,2,...,N \end{aligned}$$

10 $$\begin{aligned}&Linear \ difference \ phase:\qquad \phi _i = \sum \limits _{k=1}^i \frac{2 \pi \cdot k}{N} = \frac{i(i+1)\pi }{N} \qquad i=1,2,...,N \end{aligned}$$

where *rand*(0, 1) stands for a random number generator which returns uniformly distributed numbers in the interval [0,1]. We propose here an *iterative phase optimization* process, which competes with the linear difference phase and the other methods reported by Deng et al.^42^. In the proposed approach, it is not possible to define the phases of the different oscillations all at once, by a single equation as in Eq. 9 or Eq. 10. Phases need to be carefully selected one-by-one following an iterative procedure, except for $\phi _1$ which can be set to zero. In particular, to determine the value of $\phi _i$, the signal $S_{i-1}$ should be computed first and then M possible signals are calculated, using M different values of the $\phi _i$ parameter:

11 $$\begin{aligned} S_{i}(t,\phi _{1,...,i}) = s_i(t, \phi _i=2 \pi j/M) + S_{i-1}(t,\phi _{1,...,i-1}) \qquad j=1,2,...,M \end{aligned}$$

where $s_i$ is a single-frequency signal at the *i*th driving frequency, while $S_{i}$ and $S_{i-1}$ are the sums of the first *i*th and $(i-1)$th oscillation frequencies, respectively. Once the maximum amplitude of the obtained M signals is known, the phase value yielding the minimum signal amplitude is selected as $\phi _i$, and it is then possible to calculate the following phase-term.

**Figure 1:**
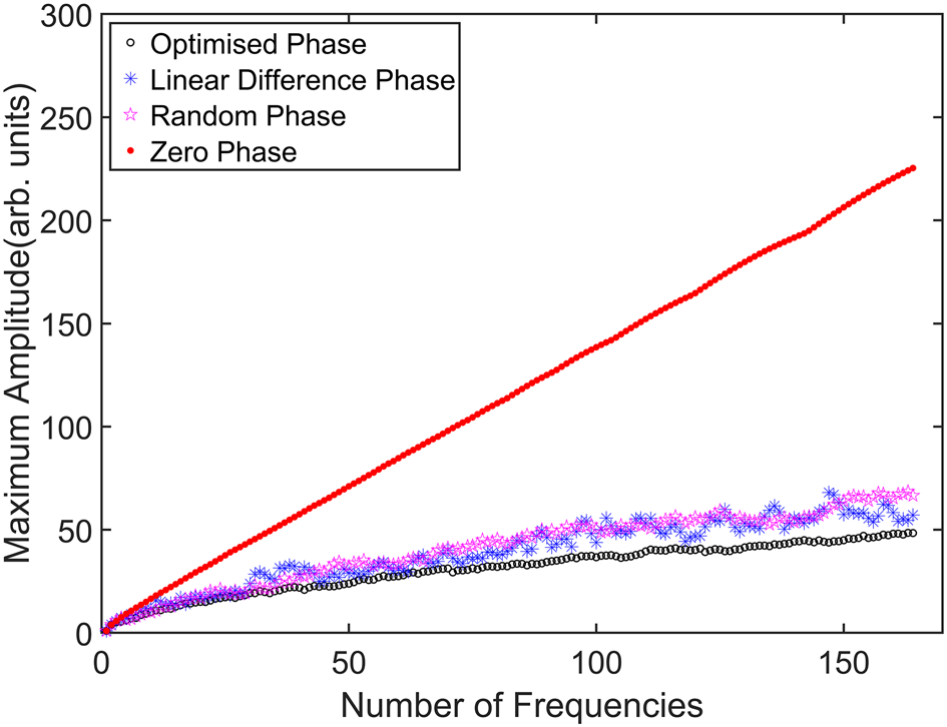
Comparison of different phase design approaches for the minimization of the maximum signal amplitude. All the prime numbers in the range [10,1000] were used as driving frequencies.

In order to compare the different approaches, the maximum amplitude as a function of the number of frequencies considered in the driving signal is evaluated. The simulated results are shown in Fig. 1. The duration of the driving signal $S_N$ was set to 1 s, sampled at a rate of 10 kHz. A step size for $\phi$ equal to 0.1 rad was selected and all the amplitudes $A_i$ were set equal to 1 without any loss of generality. Figure 1 shows the results for all the prime numbers frequencies chosen in the range $f_i \in [10,1000]$ Hz, with the initial frequency $f_1 = 11$ Hz. Here, the *zero phase* approach exhibits the worst performances with respect to the other ones, showing a linear increase of the maximum amplitude with the number of frequencies. The *Random phase* and *linear difference phase* approach exhibit similar performances but better than the *zero phase*. However, the lowest maximum amplitude is achieved using the *iterative phase optimization* approach, exhibiting the best performances among all the approaches. Hence, the *iterative phase optimization* process was adopted for the generation of the driving signals employed in the following measurements.

### Measurements on water

The combination of APC with multi-frequency stage actuation was initially validated by trapping silica microbeads with 1 μm diameter (Kisker Biotech GmbH & Co. KG, PSI-1.0) in a Newtonian fluid, as water. Silica microbeads are highly biocompatible^43^, stable in both water and organic solvents^44^ and in addition, they can be easily functionalized, thus making them a standard tool in biomedical studies^43^. The trapping efficiency, which is defined as trap stiffness per mW of laser power decreases with increasing microbead size. Higher trapping efficiencies imply smaller laser powers at equal trap stiffness and therefore less laser induced heating and photo damage. For this reason smaller particles whose diameter however are still larger than the trapping laser beam waist^45^, should be preferred over bigger particles. In our experiments, we typically choose beads of 1 μm diameter in order to have stable trapping condition.

**Figure 2:**
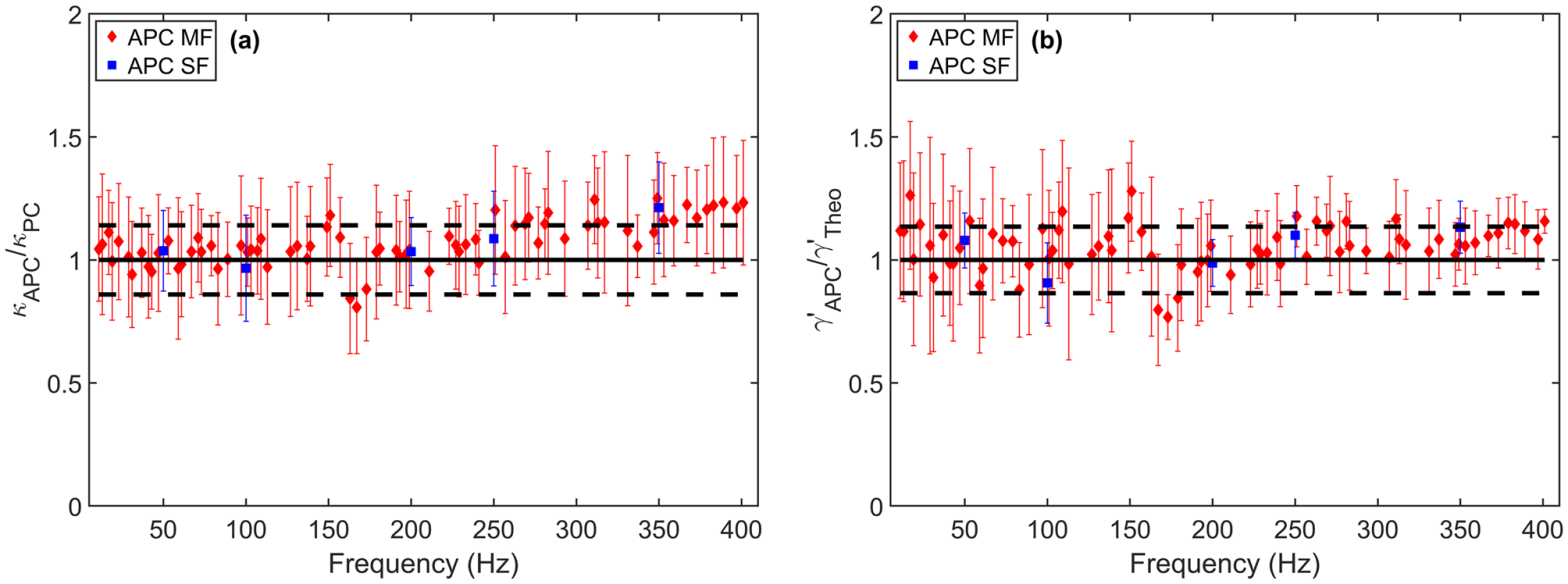
Results from measurements performed with 1 μm-diameter silica beads in water. (**a**) Trap stiffness $\kappa _{APC}$ obtained by APC compared with the reference trap stiffness $\kappa _{PC}$ derived with the PC as a function of the driving frequency. Multi-frequency measurements with prime numbers are compared to consecutive single-frequency measurements. (**b**) Real part of the friction retardation spectrum $\gamma '_{APC}$ resulting from single- and multi-frequency APC measurements compared with the theoretical value from Stoke’s law $\gamma '_{Theo}$.

Measurements in water allow us to compare APC measurements (with both single-frequency (SF) and multi-frequency (MF) actuation) with the reference standard used for passive calibration, i.e., the power spectrum method. Measurements were carried out at room temperature for nine different microbeads, and the investigated frequency span ranged from 10 to 400 Hz. In the case of multi-frequency driving, only “prime-number” frequencies were selected allowing to measure $N = 74$ frequencies simultaneously. In order to evaluate the APC performances, both single-frequency and multi-frequency measurements were performed on different microbeads and the results are shown in Fig. 2a. The ratio of the trap stiffness obtained from APC measurements—$\kappa _{APC}$—over the one derived from the power spectrum method—$\kappa _{PC}$—is plotted with respect to the driving frequency. Passive calibration (PC) by the power spectrum method was realized using the *TweezerCalib v2.0* Matlab package^46^. The black dashed lines represent the PC uncertainty, whose sources are mainly due to temperature and bead size uncertainty. The red and the blue error bars account for the standard deviation of all measurements for multi-frequency and single-frequency driving, respectively. As the power spectrum method represents a well-established calibration technique, the deviation from the unity can be used to assess the APC performances. Two primary considerations can be inferred by comparing the different calibration procedures. Firstly and most importantly, the results from PC, APC with consecutive single-frequency measurements and APC with a multi-frequency signal actuation are compatible. Secondly, both the single-frequency measurements and the multi-frequency measurements reveal the same frequency-dependence since they both show a slight increase of the trap stiffness above 200 Hz. As both measurements indicate this trend, we therefore, rule out a multi-frequency artifact.

Another parameter that can be derived using the APC approach is the friction retardation spectrum ${\tilde{\gamma }}(\omega )$, whose imaginary part is zero for a purely viscous fluid such as water. Its real part $\gamma '_{APC}$ is expected to match the theoretical value $\gamma '_{Theo} = 6 \pi \eta R$ which is defined accordingly to Stoke’s law for spherical microbeads in a viscous fluid, where $\eta$ is the fluid viscosity and R is the radius of the microbead. Figure 2b shows the comparison between the experimentally derived $\gamma '_{APC}$ and the theoretical value $\gamma '_{Theo}$ for driving frequencies between 10 and 400 Hz. The black dashed lines represent the uncertainty of the theoretical value, which is mainly due to temperature and bead size uncertainty. In accordance with the results for the trap stiffness, experimental and theoretical values of the friction retardation spectrum agree within error bars for both the single-frequency and the multi-frequency driving signals.

### Measurements on glycerol-water and methylcellulose-water solutions

As previously described, the cellular environment consists of complex fluid that exhibits both viscous and elastic properties. Our ultimate goal is to measure rheological properties in living cells, so after validating the combination of multi-frequency stage-driving and APC technique in the water, we tested our system with a more viscous (Glycerol) and a viscoelastic material (methylcellulose). The measurements were carried out at room temperature using 1 μm-diameter silica beads in glycerol-water (two different concentrations: 50 and $70\%$; $70\%$ glycerol means $70\%$ of glycerol and $30\%$ of water) and methylcellulose-water (four different concentrations: 1, 3, and $5\%$; $1\%$ methylcellulose means $1\%$ of methylcellulose and $99\%$ of water) solutions, Sigma-Aldrich methylcellulose M7140. For glycerol-water solutions measurements, two different sets of frequencies were used to demonstrate the validity of the proposed system for different combinations of driving signals. For the $50\%$ solution, every second prime number in the frequency range from 11 to 389 Hz was used, while, for the $70\%$ solution, every second prime number in the frequency range from 13 to 397 Hz was selected.

**Figure 3:**
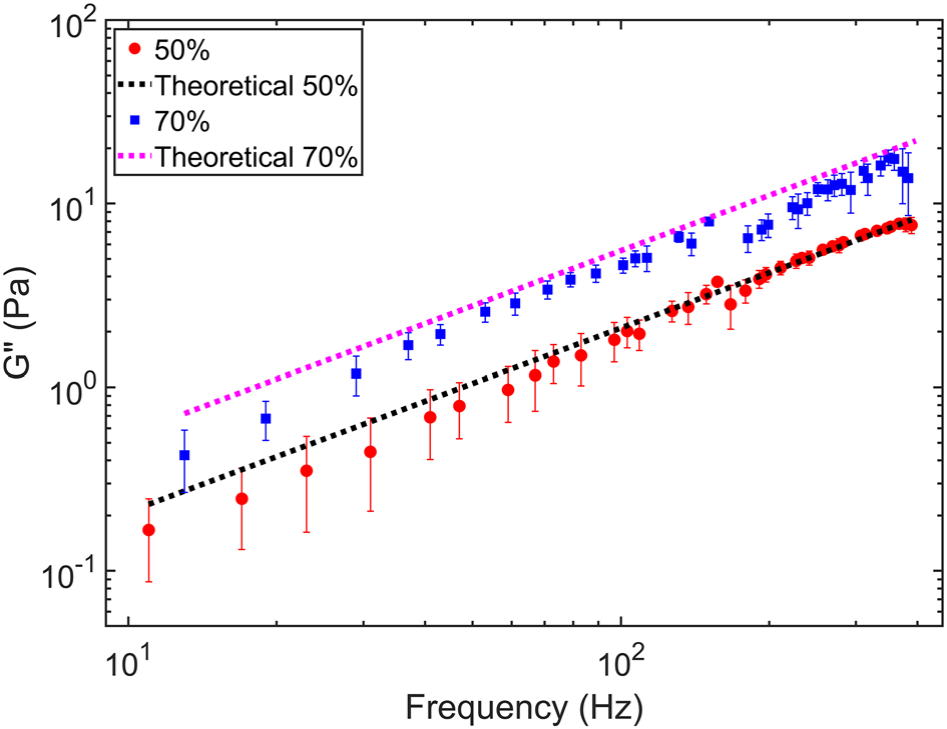
Imaginary part of complex modulus of the glycerol-water solution as a function of the driving frequency estimated by using the multi-frequency APC technique. The loss modulus of glycerol-water solutions scale as $G''\sim f^{1}$. Dotted lines show the theoretical values calculated using the Stoke’s law for spherical particles for two different concentrations $50\%$ and $70\%$.

We obtained the complex modulus, as a function of the driving frequency, calculated according to Eq. 5. The results for glycerol are reported in Fig. 3. The reported results were obtained by averaging over eighteen measurements. Measurements were performed with nine different microbeads, and for each microbead two sets of measurements were performed. The measurement uncertainty for each frequency is indicated by the corresponding error bar. Figure 3 shows the loss modulus for glycerol-water solution which scales as $G''\sim f^{1.05}$ for $50\%$ and $70\%$. This is in line with the frequency-scaling expected from the theory^47^ given by $G'' \sim f^{1}$. These measurements also match well with the theoretical value calculated using Stokes’s law for a spherical particle, shown by dotted lines. However, at low frequencies, we observe a slight deviation from the theoretical prediction. This is because the measurement values at low frequencies are largely affected by the prominent low-frequency noise contributions from the optical trap. We also observe an unusual jump in the value of the complex modulus around 150 Hz driving frequencies. This jump is consistent in all the measurements and can also be observed in Fig. 2. The possible reason for this feature is attributed to a resonance of the driving stage. A thorough investigation of this resonance phenomena will be carried out in future. For this reason, we ignored the frequencies in the range from 150 to 200 Hz in the following measurements with methylcellulose.

**Figure 4:**
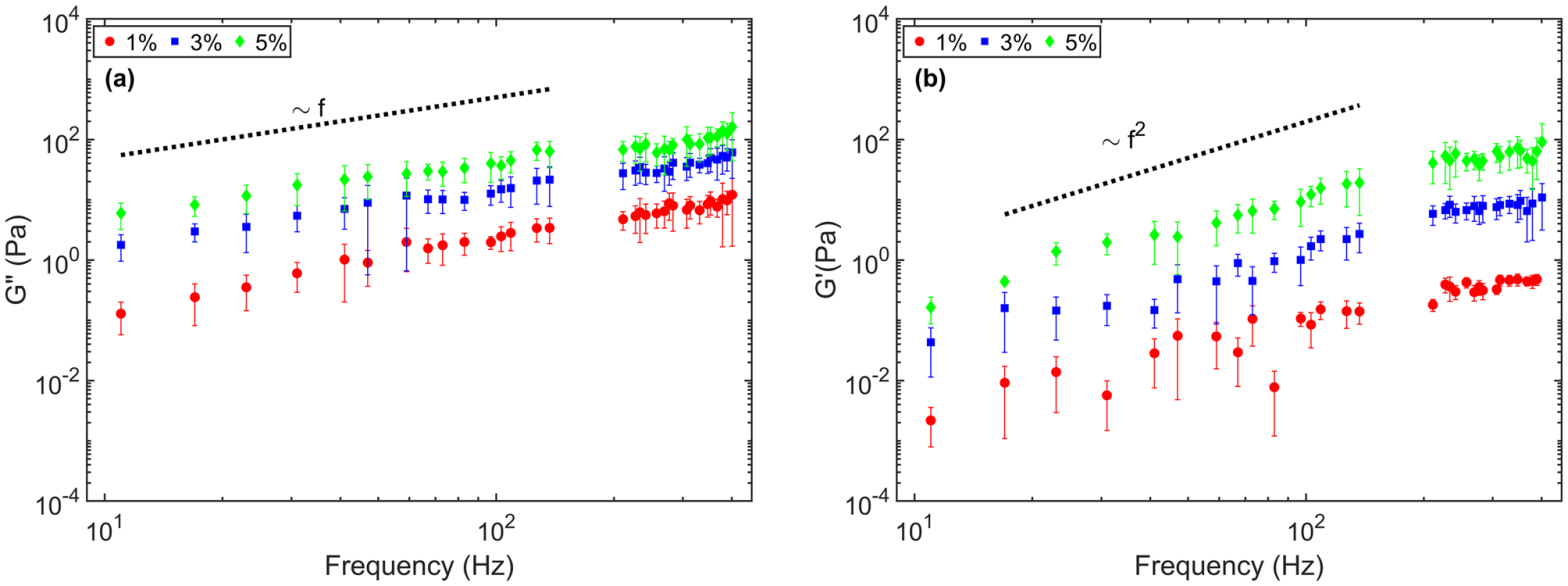
Complex modulus of the methylcellulose-water solution as a function of the driving frequency estimated by using the multi-frequency APC technique. (**a**) loss modulus of 1, 3 and $5\%$ methycellulose-water solutions and (**b**) storage modulus of 1, 3 and $5\%$ methycellulose-water solutions. Theoretically the Loss modulus scale as $G''\sim f^{1}$ and the storage modulus scale as $G' \sim f^{2}$ indicated as black dotted lines.

After performing measurements with glycerol, we carried out measurements in viscoelastic methylcellulose-water solutions. The loss and storage moduli resulting from the measurements of the different methylcellulose-water solutions are shown in Fig. 4a,b, respectively. The loss modulus for the solutions scales approximately as $G''\sim f^{0.9}$. This trend is in line with the expected behavior according to polymer theory^48^, which is given by $G''\sim f^{1}$ (see black dotted line in Fig. 4a. The storage modulus is expected to scale as $G' \sim f^2$ as shown by the dotted line in Fig. 4b) and a good agreement was found between the experimental results and the predicted scaling up to around 300 Hz. However, for larger frequencies, the experimental data for storage modulus scales as $G' < f^2$. The drop in the power coefficient at higher frequencies results from the shear based thinning of methylcellulose. Shear thinning is a well-known non-Newtonian property of fluids whose viscosity decreases under an increased shear strain^49–53^. Ghannam and Esmai^50,51^ have shown that the shear thinning depends on the concentration of carboxymethyl cellulose (CMC). Similarly, our measurements showed a similar trend for different concentrations of methylcellulose.

## Discussion

APC represents a very promising technique for the calibration of OT in viscoelastic materials allowing to determine both the trap stiffness and the rheological properties of the medium. Laser driving is usually preferred with respect to stage driving for active measurements due to the possibility of reaching higher oscillation frequencies. On the other hand, the use of two lasers in laser driving experiments, a trapping laser and a detection laser, make the experimental setup implementation more complicated. Despite the more straightforward implementation, to the best of our knowledge, APC with single-frequency stage driving for rheological applications has been demonstrated up to a maximum frequency of 100 Hz^28,54^. In this work, we present the results of microrheological experiments performed by an improved version of the APC technique with multi-frequency piezo-stage actuation. Measurements have been performed in water, glycerol-water and in a methylcellulose-water solutions using 1 μm diameter silica microbeads as probes. We demonstrated the possibility of carrying out broadband APC experiments with stage actuation from 10 to 400 Hz. In addition to the significant bandwidth increase, the use of a multi-frequency driving signal reduces the time for measuring all the frequency-span to just a few seconds. Such a result is obtained thanks to the accurate optimization of the stage-driving signal, which includes only prime-number frequencies, to avoid any cross-talk in the frequency domain, each of them with a carefully selected phase. We presented an *iterative phase optimization* method for multi-frequency signal creation which allows generating driving signals with a low maximum amplitude. This aspect has two advantages with respect to techniques previously reported in the literature. Firstly, the requirement concerning the low-amplitude of the particle oscillation imposed by Onsager’s regression hypothesis is met even with a large number of frequencies driven simultaneously, allowing the measurement over a wide-frequency band. Secondly, a minimized oscillation amplitude is particularly indicated when heterogeneous biological samples are investigated in order to determine rheological properties with a high spatial resolution and without damaging the internal structure of the living object. Moreover, the reduced measurement time gives the possibility of characterizing the frequency-dependent viscoelastic properties of biological samples such as cells, otherwise impossible to be studied with longer single-frequency measurements, in which case cells may self-reorganize and change their properties.

In conclusion, the optimized APC protocol presented in this paper can be employed for high temporal and spatial resolution measurements in vivo over a wide frequency range and with the less-demanding experimental implementation of the stage driving approach.

## Methods

### Water-methylcellulose solution preparation

**Figure 5:**
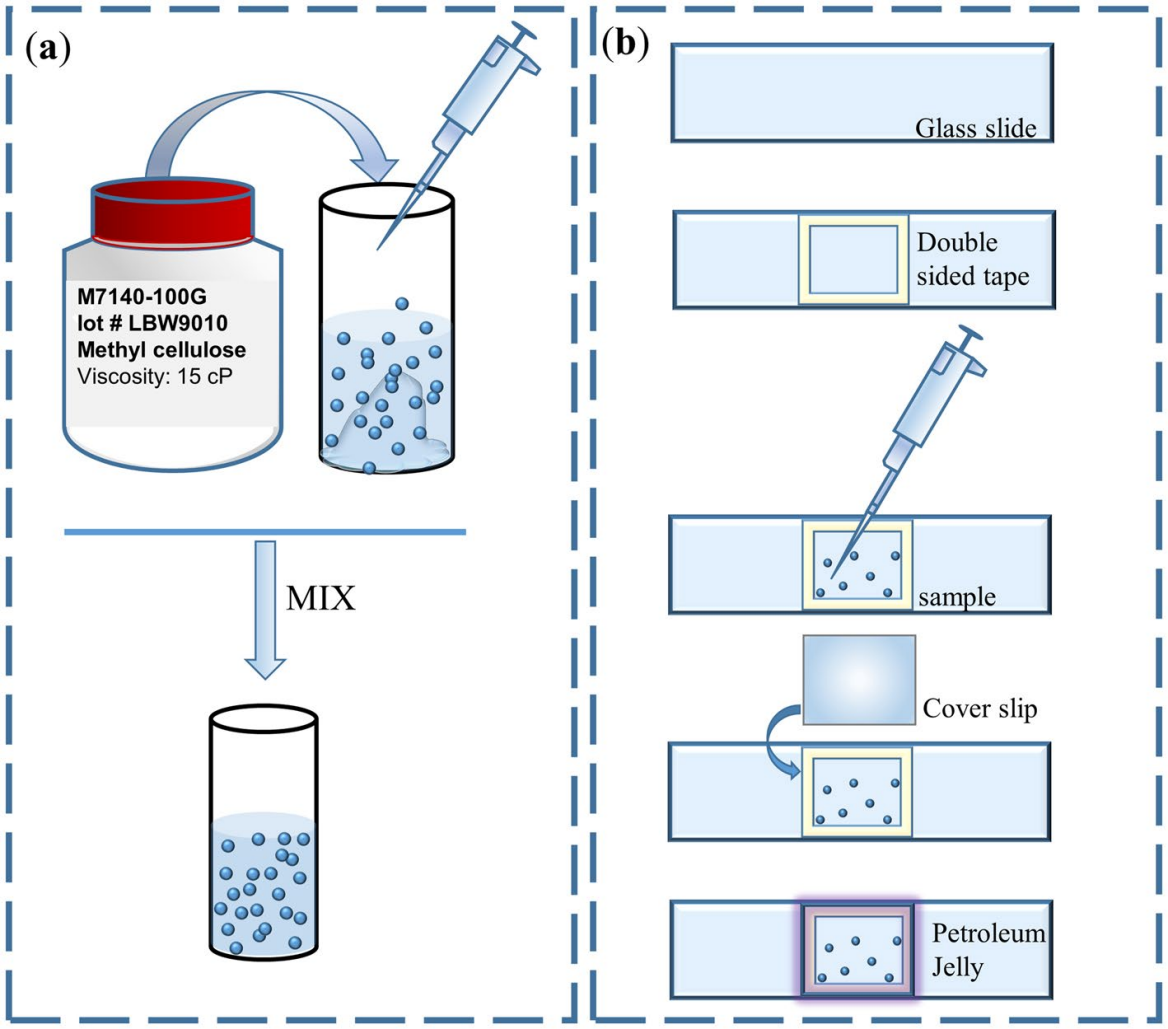
(**a**) Preparation of the methycellulose solution, (**b**) closed-chamber sample preparation.

Methylcellulose-water solutions at different concentrations were prepared by dissolving methylcellulose powder (Sigma-Aldrich, methylcellulose M7140) in water, previously heated to $80 \ ^\circ$C. 0.5 μL of 1 μm diameter silica microbeads (Kisker Biotech GmbH & Co. KG, PSI-1.0), per milliliter of methylcellulose-water solution were added to it. The solution was then gently mixed and was let to thermally equilibrate to room temperature before curing overnight at $5 \ ^\circ$C. Solutions at concentrations equal to 1%, 3%, 5% (w/v) were prepared and characterized. A sketch of the preparation of methylcellulose solutions is shown in Fig. 5 (a).

### Closed-chamber sample preparation

A closed-chamber geometry was proposed and tested for the sample preparation in order to minimize the hydrodynamic effects. The sample preparation consists of different steps, graphically shown in Fig. 5b. A 1 mm-thick glass microscope slide (Carl Roth by Menzel, hydrolytic class 3) is used as the sample basis. These are standard microscope slides which are chemically inert when used for aqueous media at moderate temperature. Then, a double-sided sticky tape (Tesa) is cut into the desired border shape and placed onto the microscope glass slide. After that, the sample solution is injected with a standard micro-pipette before closing the chamber with a coverslip (Carl Roth, hydrolytic class 1, thickness 0.13–0.16 mm). While closing the sample chamber with coverslip, special care is taken to avoid air inclusion as this would lead to a decoupling of the fluid and the chamber wall, which may result in an underestimation of the perturbation magnitude. In addition the border is sealed with petroleum jelly in order to increase the bonding between the cover slip and the microscope slide to prevent sample evaporation. Finally, the sample is mounted on to an inverted microscope OT setup and measurements are recorded and stored on a computer.

### Experimental setup

**Figure 6:**
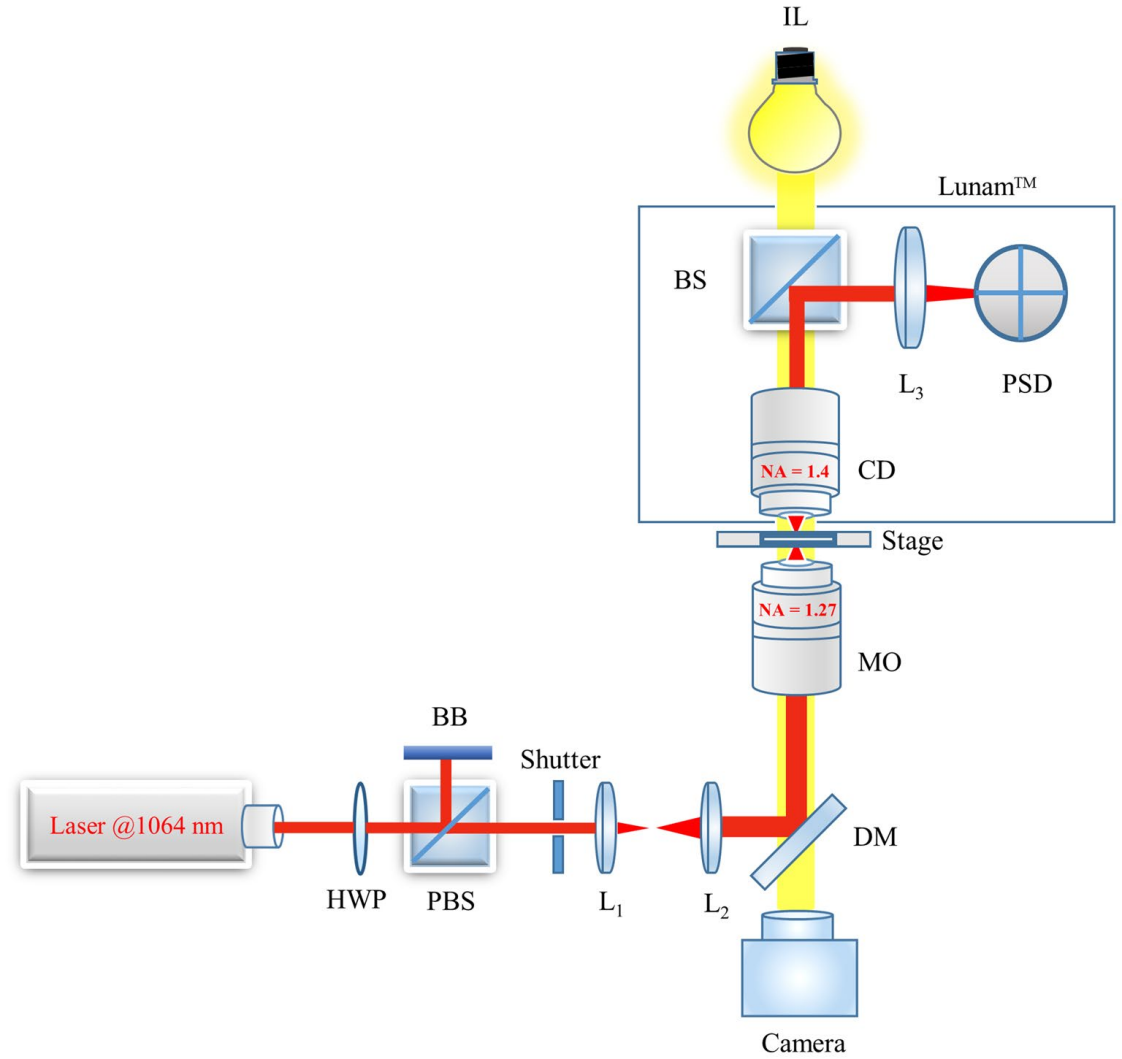
Schematic representation of the stage-driven OT setup with a position-sensitive detector in the back focal plane. An inverted microscope has been extended by both a commercially available back-focal-plane interferometer and a piezo nano-positioning stage. A continuous-wave diode-pumped laser emitting at 1064 nm is employed for optical trapping. *HWP* half wave plate, *PBS* polarizing beam splitter, *BB* beam blocker, *Li* Lens, *DM* Dichroic mirror, *MO* microscope objective, *CD* condenser, *BS* beam splitter, *PSD* position sensitive device, *IL* illumination lamp.

The schematic of the experimental setup is shown in Fig. 6. A continuous-wave (CW) diode-pumped laser operating at a wavelength of 1064 nm (Cobolt Rumba) is employed as an optical source. The laser emits a high quality ($M^2 < 1.1$) single Gaussian $TEM_{00}$ mode with a maximum output power of 3 W. A half-wave plate (HWP) placed in cascade to a polarizing beam splitter (PBS) allows manually to regulate the intensity of laser light in the trapping plane. A shutter (Uniblitz LS3Z2) can be used to block the laser radiation, in case it is needed. Two lenses ($L_1$ and $L_2$) expand the laser spot to slightly overfill the back aperture of the objective in order to achieve a higher trapping efficiency. The laser radiation is guided by a dichroic mirror (DM) into an inverted microscope (Nikon Eclipse Ti-E) which is equipped with a 60x water-immersion objective (Nikon CFI Plan Apo IR SR 60XWI) with a numerical aperture of 1.27. The objective lens is designed to minimize aberrations while providing a long working distance of 0.17 mm which allows trapping within a thicker specimen. A commercially available Lunam T-40i (Impetux Optics, Spain) is used as a back-focal-plane interferometer (BFPI) for position detection measurements with less than 1 nm spatial resolution. The Lunam consists of an oil-immersion condenser (CD) lens with a numerical aperture of 1.40. This system can even be used for direct force^55^ measurements with a resolution of 10 fN extending the flexibility of the setup for in vivo measurements. Besides, a CMOS camera (UI-3370CP-M-GL) is mounted on the side port of the microscope to observe the trapped microbead. In order to perform APC measurements, the setup is provided with a nano-positioning piezo stage (Aerotech QNPHD30L-10) in combination with a four axes piezo motor controller (Aerotech Ensemble QLAB). The piezo stage is driven by a function generator (Agilent Technologies, USA), it has a sub-nanometer resolution (0.02 nm Open-Loop) and it is designed for high-speed applications. Characterization of the performance of the piezo stage under load was initially carried out in order to assess the frequency-dependence of the stage driving. As expected, the amplitude of the stage oscillations resulted in being a decreasing function of the driving frequency. Specifically, the piezo stage behavior can be described by a third-order low pass filter with a cut-off frequency equal to $\sim 300$ Hz. A correction factor for the frequency-dependence of the piezo stage has been included in the driving signal generation in order to have a microbead oscillation amplitude constant over the whole frequency range for the multi-frequency APC.

### Devices synchronization

The trapped microbead position is acquired by the Lunam which is triggered by a signal generated by a NI box (National Instruments NI USB-6251 BNC) and its data are digitally read out by a computer. The stage amplitude signal, instead, is analogically acquired by the NI box, which is connected to the computer. A synchronization measurement is therefore necessary in order to determine the time delay between the acquired signals. This calibration step is crucial, as a fixed time delay (like the one that has been experimentally determined) can result in a frequency-dependent phase lag and, therefore, a wrong estimation of the complex quantities involved in the APC measurements. For the determination of the time delay, a further experiment can be performed consisting of sinusoidally oscillating a microbead stuck onto the chamber’s bottom surface. The microbead will thus move synchronously with the piezo stage and this allows determining the phase delay between the microbead position signal acquired by the Lunam system and the piezo stage amplitude signal recorded by the NI box. From the phase difference $\Delta \phi$ between the two signals it is possible to determine the time delay as $\Delta t = \Delta \phi / (2 \pi \cdot f)$ with *f* being the oscillation frequency. Parallel measurements can be performed by a multi-frequency actuation of the sample stage with a proper driving signal. By analyzing the acquired signals in the Fourier space, it is possible to derive the time delay independently for each frequency. A linear increase of the phase delay with respect to the driving frequency has been found, resulting in an estimated fixed time delay $\Delta t \sim 114\pm 9$ μs. Calibrations of the time delay have been carried out on different days with no modification in the experimental setup yielding repeatable results.

### Position sensitive detector calibration

As discussed in the previous paragraph, a signal proportional to the microbead position is acquired by the Lunam. However, the recorded data have the unit of measure of force, [N], as the Lunam is designed for direct force measurements. Therefore, a calibration constant $\beta$ has to be determined in order to retrieve the real microbead position in [m] from the acquired data^25^. In case of a viscous fluid of known viscosity, such as water (as discussed in the “Measurements on water” section), the $\beta$ calibration can be performed considering the measured thermal power spectrum which has been introduced in Eq. 1. The power spectrum of a spherical particle with radius *R* in a viscous medium of viscosity $\eta$ is given by a *Lorentzian* function:

12 $$\begin{aligned} P_U(\omega ) = \frac{2 D}{\omega _c^2 + \omega ^2} \end{aligned}$$

where $\omega _c = \kappa / \gamma _0$ is the corner angular frequency, $\gamma _0 = 6 \pi \eta R$ is the friction coefficient and $D = k_B \cdot T / \gamma _0$ is the diffusion coefficient given by the Einstein relation^56,57^. By fitting the experimental Brownian motion of a trapped microbead to the Eq. 12, the unit of measure of the fitted diffusion coefficient $D_{fit}$ will then be $[N^2/s]$. By comparing the experimentally determined diffusion coefficient, $D_{fit}$, with the theoretical one given by the Einstein relation, *D*, it is possible to determine the position calibration factor as:

13 $$\begin{aligned} \beta = \sqrt{\frac{D}{D_{fit}}} \end{aligned}$$

which has the unit of measure of [*m*]/[*N*]. In the case of a viscoelastic material of unknown rheological properties (as discussed in the paragraph Measurements on methylcellulose-water solution), a further measurement in addition to the passive and active experiments has to be performed in order to determine $\beta$. In this case, the protocol for the position-sensitive detector calibration follows different steps. A microbead stuck to the chamber bottom surface is selected and the trapping laser spot is positioned over the microbead central position. The piezo stage is then driven sinusoidally at low frequency (e.g., 5 Hz) and both the stage position signal ([*m*]) and the Lunam signal ([*N*]) are recorded. The stage position signal exactly describes the microbead position, as the microbead is stuck to the chamber bottom surface. Finally, by comparing the two signals in the linear response region of Lunam, it is possible to find the calibration factor $\beta$. The determination of calibration factor $\beta$, using this method has the advantage that it does not require the knowledge of experimental parameters such as the radius of the bead and the rheological properties of the medium. Therefore, this method is applicable both for viscous and viscoelastic materials. In case of water, we determined the calibration factor using both methods and the results were comparable.
